# Stigmatization and Self-Perception regarding issues related to Mental Health: A qualitative survey from a lower and middle-income country

**DOI:** 10.12669/pjms.39.5.7487

**Published:** 2023

**Authors:** Shaffaque Ikhtiar Khan, Muhammad Irfan

**Affiliations:** 1 Shaffaque Ikhtiar Khan, House Officer, Riphah International University, Islamabad, Pakistan; 2 Prof. Dr. Muhammad Irfan, Department of Mental Health, Psychiatry and Behavioural Sciences, Peshawar Medical College, Peshawar, Pakistan

**Keywords:** Mental health, Stigma, Noncompliance

## Abstract

**Objective:**

To assess the understanding of the patients with common mental disorders, towards issues related to their mental health.

**Methods:**

This qualitative study was conducted from December 2018 to April 2020. Thirty-four patients, suffering from common mental disorders, were interviewed in public and private sector hospitals of Peshawar. The interviews were recorded, transcribed, translated into English, and themes were generated from their responses. Content analysis was carried out on the data obtained. The themes resulting from each interview were then further comparatively analyzed.

**Results:**

The mean age of the sample was 31.9±10.61 years. Most of the patients (n=24, 70.6%) were aware that the nature of their illness was a psychological one with a majority (n=17, 50%) describing it with the symptoms of headache or burden on the head. Most of the patients (n=14, 41.1%) were unaware of the general public opinion towards mental disorders but those who were aware described these with stigmatizing descriptions e.g., “people call them crazy” etc. Most of the patients (n=20, 58.8%) were unaware about their own opinion regarding their illness and some said that they tried to conceal their illness from others. Unfortunately, most of the patients (n=19, 55.8%) were not aware of mental healthcare professionals or the existence of psychiatry as a profession.

**Conclusion:**

Stigma, both public and personal, was quite high, which caused patients to feel compelled to conceal their illness. There was also a general lack of knowledge with regard to mental disorders in our society. The general public opinion about mental health professionals was not favorable.

## INTRODUCTION

“Mental health is a state of well-being in which an individual realizes his or her own abilities, can cope with the normal stresses of life, can work productively and is able to make a contribution to his or her community”.^1^ Mental health is important for emotional expression, social interaction, and economic independence, both individually and collectively.^2^ According to the Biopsychosocial model, an individual’s social, psychological, and biological factors determine their level of mental health and a decline in any or all of these factors may result in a mental health issue. People with mental health issues experience an inability to form healthy relationships which may cause impaired quality of life, that could ultimately lead to suicide.^3^ According to the World Health Organization, mental disorders are said to be the foremost cause of disability affecting over 450 million people all over the world.^4^

Despite their implications, these mental disorders are often left undiagnosed, mainly because of the reluctance of people to acknowledge and seek help for their disorders, the key reason for which is the stigma associated with the word “mental illness”.^5^ Stigma, which is defined as a mark of disgrace associated with a particular circumstance, quality, or person; may be of two types i.e., public stigma and personal stigma.^6^ Self-stigma and anticipated/expected stigma are two subtypes of personal stigma.^7^ Anticipated stigma in people with mental disorders is the belief that others in society will think negatively or inferiorly about them, and self-stigma is the internalization of these prejudices. In self-stigma, the person labels themself as inadequate and socially unacceptable. People suffering from personal stigma refuse to see their symptoms as a possible mental problem and fail to seek professional help for it.^7^

The knowledge a person has towards the existence of mental health disorders can play a role in their decision to seek treatment. However, knowledge about mental health is quite insufficient, and this leads to a lot of misconceptions and cultural beliefs that are inaccurate and not based on any real facts,^8^ Consequently, people seek other sources for treatment rather than consulting a professional.^9^ This study, therefore, focuses on attitudes and general awareness in the community regarding mental disorders. It also focuses on both personal and public stigma, associated with mental disorders, and how all these collectively play a role in creating misconceptions towards mental healthcare and a reluctance towards seeking professional help for mental disorders. Our objective was to assess the understanding of the patients with common mental disorders, towards the issues related to their mental health**.**

## METHODS

This qualitative research was conducted in public and private sector hospitals of Peshawar from December 2018 to April 2020. Thirty-four patients from the psychiatric units, with common mental disorders were recruited in the study. Each patient was approached for permission and informed consent was taken.

### Ethical Approval:

Ethical Review Committee of the institution approved the study (No:12^th^-UMR-022 on 05-01-2019).

This study was conducted in the form of one-on-one interviews using a flexible topic guide covering important areas, developed by following the protocol and was validated by the experts. The researcher interviewed patients with regards to their knowledge concerning their illness and personal/community attitudes towards mental disorders. They were asked about cultural beliefs, and how these play a role in their reluctance towards seeking professional help and support for their illness.

The interviews were audio-recorded, transcribed, translated into English, and themes were generated from their responses. The themes resulting from each interview were then further comparatively analyzed and used to explain the role that the lack of knowledge and associated stigma, both public and personal, play in reluctance towards seeking professional help in people with mental health issues. Thematic analysis was used, using Virginia Braun and Victoria Clarke six steps approach to generate themes from the qualitative interview data. The six steps included familiarization with the data, generating initial codes, searching for themes followed by reviewing, understanding, naming the themes, and finally producing the results.

## RESULTS

The mean age of the sample (n=34) was 31.9 ± 10.61 years. Out of 34 patients, 22 (64.7%) were females. Half the patients (n=17, 50%) received education till high school or higher and 18 (52.9%) were married. The majority of the patients (n=22, 64.7%) were unemployed at the time of the study. Table-I describes the patient characteristics in detail.

**Table-I:** Profiles of the participants of the study.

S. No	Variable		n (%)
1	Gender	Male	12 (35.3%)
		Female	22 (64.7%)
2	Age Range (in Years)	<20	6 (17.7%)
		20-39	19 (55.9%)
		40-59	8 (23.5%)
		≥60	1 (2.9%)
3	Highest Education	No formal Education	16 (47.1%)
		Primary School or Lower	1 (2.9%)
		High School	14 (41.2%)
		College/ University	3 (8.8%)
4	Marital Status	Single	16 (47.1%)
		Married	18 (52.9%)
5	Employment Status	Unemployed	22 (64.7%)
		Employed	12 (35.3%)
6	New/ Old Patient	First Visit	19 (55.9%)
		Follow Up	15 (44.1%)

***The following five themes emerged during the qualitative interview:***

***Theme-1: Impact of hierarchy of labels:*** This theme captured the patient’s perceptions regarding the impact and effect of the ‘hierarchy of labels’ on themselves. When we asked them to describe their symptoms, most of the patients (n=24, 70.6%) were aware that the nature of their illness was a psychological one. However, a good number of the patients (n=10, 29.4%) were unaware of what it was.

Following were their responses.

*“I face problems at home and have no control over my nerves.” (P-5)*

*“I feel like someone is coming after me to kill me, and then I faint.” (P-10)*

*“I feel restless, negative thinking basically.” (P-18)*

*“A feeling of suffocation, I don’t feel like going anywhere to any events.” (P-30)*

*“My heart feels sad all the time” (P-21)*

*“There is always a burden on my head.” (P-12)*

Some patients had a very unique way of describing their illness and the cause behind it, for example, one of them described the cause of their illness as follows,

“*It’s because of laughing too much” (P-19)*

We also explored how patients described their symptoms and majority of the symptoms experienced by the patients were headache/burden on head (n=17, 49.9%); followed by Sadness (n=8, 23.5%); anger (n=6, 17.6%); tension, insomnia and negative thoughts (n=5, 14.7%, each). Few of the patients reported body aches (n=4, 11.7%), fainting, nausea/loss of appetite, feeling suffocated (n=3, 8.8%, each) and fearfulness (n=2, 5.8%). Only 1 (2.9%) reported experiencing crying and being restless.

***Theme-2:*** Mental illness, a stigma that shames us all:

The respondents were asked about the public stigma towards mental illnesses and the social stereotypes that they have to face. Most of them were unaware of it, however, those that were aware described general public opinion about people with mental illness as follows.

*“People laugh at them and call them crazy; they say they’re psycho, the disease itself is a stigma” (P-1)*

*“Only crazy people go to the psychiatrist” (P-31)*

*“They don’t consider it an actual illness; they think its black magic” (P-32)*

*“Relatives don’t like to keep any relations with those having mental illnesses” (P-31)*

*“No one wants to wed their daughter off to someone that is psychologically ill” (P-7)*

*“People don’t care about the sickness or the sick, they consider that it happens to get attention.” (P-34)*

*“They show a lot more sympathy towards people who are suffering from other non-mental health-related issue.” (P-33)*

*“When people find out about someone having mental health problems, they say stuff like God is punishing them or that they are sick because they are sinners. Or that they have bad intentions towards others which is the reason why they are sick.” (P-34)*

We asked the patients about public opinion about people with mental health disorders and their responses are tabulated in Table-II.

**Table-II:** General public opinion about people with mental health issues.

S. No	Response	Number	%
1	No Idea	14	41.2
2	Call them crazy	11	32.4
3	Call them mentally ill	3	8.8
4	Think that they fabricated their illness	2	5.9
5	Have negative feelings towards them	1	2.9
6	Call them “tensioni”	1	2.9
7	Think that mental illnesses are untreatable	1	2.9
8	Say that their mind is weak	1	2.9

***Theme-3: Personal Stigma:*** During the interview, each patient was asked for their opinion about their own illness. Most of them (n=20, 58.8%) were unaware or had no opinion about it. Some patients said they tried to conceal their illness from others. Their personal stigma was obvious from the following narratives.

*“I just tell everyone that I have a heart problem when someone asks.” (P-30)*

*“I don’t tell people because when people find out they say bad stuff like God is punishing me or that I am sick because I am a sinner.” (P-32)*

*“I don’t tell people because when I did, I was told that since I had bad intentions towards others, that is the reason why I was not well.” (P-34)*

***Theme-4: Seeking Help:*** The respondents were asked if they themselves were willing to seek psychiatric help and most of them (n=20,58.8%) showed willingness towards seeking help, whereas (n=2,5.8%) patients were not willing and had to be brought to the hospital by someone else. Almost one-third (n=12, 35.2%) were not open to responding about it, regarding compliance issues of the willing patients, the following were their responses.

*“I don’t want to stop treatment from this doctor but my family doesn’t bring me, as we are poor and we don’t have any money.” (P-6)*

*“For my follow-up care, I have to beg my husband” (P-11)*

***Theme-5: Knowledge about mental health support network:*** Most (n=19, 55.8%) of the patients were not aware of mental healthcare workers or even the mere existence of such a profession. Those who had some idea (n=10, 29.4%), responded that mental health professionals treat the head/brain/mind. Only 3 (8.8%) of the respondents had knowledge about psychiatrists, their profession, and their job description. One of them described layperson description of psychiatrists as follows,

*“People don’t have a very good opinion about psychiatrists. They say only people who are completely crazy and mental, seek their help. They also say that psychiatrists themselves are crazy” (P-31)*

## DISCUSSION

Mental health, unfortunately, is a very neglected field of healthcare in Pakistan and even the chances of improvement are exceedingly bleak as most affected individuals never seek treatment, or don’t follow through with their treatment due to stigma. A major reason for stigma can be a lack of knowledge, observed in almost all participants of our study, as very few of our patients could specify their disorder by name. Most patients had a unique manner of describing their symptoms. Some of the symptoms described in varying details were sadness, headache, tension, anger, insomnia, crying, nausea, loss of appetite and feelings of suffocation, etc. The study conducted by Liu et al reported that symptoms of mental illnesses are subjective and vary a lot from person to person, and each patient has a different manner of describing their illness.^10^ Most of our patients were aware of mental healthcare and mental illnesses whereas a similar study conducted by Mahmoud et al assessing different factors acting as barriers towards mental health treatment, revealed that out of a total of 5644 participants, 87% were lacking in awareness towards the mental healthcare services available to them.^11^

In Pakistan, like all underdeveloped countries, mental illnesses are poorly represented due to a significant stigma associated with them.^12^ People who have mental health issues suffer from both the nature of their illness, and from the societal prejudice that they have to face.^13^ As a result their quality of life is severely impacted. Rüsch et al reported that the biggest barrier towards availing mental healthcare services was stigma.^14^ The same is also reported in our study. Corrigan et al suggested that many hurdles faced by people suffering from mental illnesses were due to social stigma, hence effectively depriving them of their fair shot at a good quality of life.^15^ Similarly, Byrne et al emphasized on the shame associated with mental illnesses due to the stigma faced by individuals, which leads to secrecy that can act as a barrier towards seeking help. The study also discusses the stigma experienced by those close to the patient, simply because of their association with the patient.^16^ Phelan et al noted that among a total of 156 caretakers, half of them admitted to making efforts to hide the illness from others.^17^ However, there is a lot of work that still needs to be done to understand the degree and nature of the prejudice faced by those that are mentally ill and the impact of this prejudice on the quality of their lives.

Another impact of stigma is on the self-perception of patients with mental health issues, the likelihood of these patients internalizing the cultural prejudice that they face is great.^18^ This can have a negative impact on their confidence and sense of self-worth. In our study, patients narrated that they have to describe their mental disorders in physical terms so as to get the appropriate treatment on time. In order to get to the root of the problem and address the impact of stigmatization, it is integral to explore different opinions and perspectives of people with mental disorders, with regards to their own illness, as levels of self-stigma is reported to be higher in individuals living in countries with more stigmatizing attitudes towards mental health.^19^

Noncompliance is considered one of the commonest causes of treatment failure. In our study noncompliance was rooted in an unwillingness to seek treatment due to financial constraints i-e., low socioeconomic status. In research conducted by Rao et al, it has been shown that compliance is higher in patients with middle or higher socioeconomic status.^20^ Additionally in our study, non-compliance was higher in female patients, mostly stemming from a lack of support provided by the male members in their household that they are financially dependent upon for their livelihood. Our findings are supported by Bener et al, who stated that out of the total compliant patients, 49.4% have great family support, and Gebeyehu et al who claimed that patients with no social support have a very high nonadherence rate towards treatment.^21^,^22^

The awareness about mental health professionals in our society, though growing, is still less, and Psychiatry as a profession is also greatly stigmatized, an aspect that is supported by our study. Similarly, a study by Kareem et al showed that despite having a positive attitude towards the profession and finding it a respectful one, only 19.4% of the respondents considered it as a career option, whereas the majority of the respondents were unwilling to pursue it as a career.^23^ A study conducted by Niaz et al showed that 54% of medical students were reluctant to choose psychiatry as a career option due to the concept that psychiatrists are less respected as compared to other specialists.^24^ Additionally, a study carried out by Angermeyer et al, claims that psychiatry as a profession faces low esteem by the public and that there is a growing concern about the poor image of the discipline in the eyes of the public and medical students.^25^ All this stigma associated with the mental health profession might also act as a significant contributing factor towards a reluctance in seeking help. However, the concept needs to be explored further.

### Limitations:

The results could not be representative of the general population since the study focused on perspectives. Moreover, responses may only be considered appropriate in the cultural context and may not be generalizable to people who are socio-culturally different.

## CONCLUSION

Stigma, both public and personal was quite high, with most patients receiving comments about being crazy and some patients admitted to trying to conceal their illness from others. Additionally, there was also a general lack of knowledge with regards to mental disorders in our society. Compliance rates were high with only a few patients unwilling to seek treatment. The general public opinion about people with mental health issues and mental health professionals was not favorable, rather stigmatized.

### Authors Contribution:

**SIK** conceived the idea, conducted the interviews, and prepared the manuscript

**MI** designed the study, analyzed the data and helped in the write up of the manuscript.
